# Exploratory Longitudinal Analysis of the Circulating CHIT1 Activity in Pediatric Patients with Obesity

**DOI:** 10.3390/children10010124

**Published:** 2023-01-06

**Authors:** Ioana Țaranu, Nicoleta Răcătăianu, Cristina Drugan, Cristina-Sorina Cătană, Andreea-Manuela Mirea, Diana Miclea, Sorana D. Bolboacă

**Affiliations:** 1 Department of Medical Informatics and Biostatistics, Iuliu Hațieganu University of Medicine and Pharmacy, Louis Pasteur Str., No. 6, 400349 Cluj-Napoca, Romania; 2 Pediatric Clinic I, Emergency Pediatric Hospital, Calea Moților, No. 68, 400370 Cluj-Napoca, Romania; 3 Integrated Ambulatory of Endocrinology, Infectious Diseases Clinical Hospital, Calea Moților, No. 19, 400000 Cluj-Napoca, Romania; 4 Department of Medical Biochemistry, Iuliu Haţieganu University of Medicine and Pharmacy Cluj-Napoca, Louis Pasteur Str., No. 6, 400349 Cluj-Napoca, Romania; 5 Department of Medical Genetics, Iuliu Hațieganu University of Medicine and Pharmacy, Louis Pasteur Str., No. 6, 400349 Cluj-Napoca, Romania

**Keywords:** human chitotriosidase (CHIT1), longitudinal analysis, children, obesity, insulin resistance (IR), inflammation, puberty, abdominal obesity, 24 base-pair duplication

## Abstract

Macrophage activation and cytokine release play a pivotal role in inflammation-mediated metabolic disturbances in obesity. The proinflammatory macrophage secretes human chitotriosidase (CHIT1). The expression of the CHIT1 in visceral adipose tissue is associated with cytokine production. Our study aimed to assess whether the CHIT1 circulating activity, as a macrophage activation indicator, reflects the change of the adiposity level and the insulin resistance (IR) in children with obesity. We longitudinally (median follow-up period of 7 months; IQR [5 to 8.5] and {2 to 13} months) evaluated the CHIT1 circulating activity, the adiposity level (waist circumference (WC), waist-to-hip ratio (WHR), waist-to-height ratio (WtHR), and body mass index (BMI)-for-age z score), and two surrogate markers of IR (Homeostatic Model Assessment for Insulin Resistance, HOMA-IR and the triglycerides-to-high density lipoprotein cholesterol ratio, TG/HDLc) in 29 pediatric patients (16 girls and 13 boys) with obesity. We found a significant reduction in CHIT1 circulating activity (Wilcoxon test, *p* = 0.015) and a decrease in TG/HDLc at the follow-up evaluation (Wilcoxon test, *p* < 0.001). Indicators of adiposity were positively correlated with HOMA-IR at baseline, among which WC was the sole indicator associated with HOMA-IR (Spearman’s rank correlation coefficients, *p* < 0.05) at follow-up. Human chitotriosidase has the potential to be a valuable measure of the progression of subclinical inflammation in children with obesity. Subclinical inflammation, as expressed by the circulating CHIT1 activity, progresses independently of the abdominal adiposity, as measured by the clinical indicators, and is associated with a change in insulin resistance.

## 1. Introduction

Obesity in children is associated with increased cardiovascular risk in adulthood [1,2], with important implications for the general mortality [3,4]. In adults with obesity, molecular alterations (i.e., the activation of the endoplasmic reticulum stress response and the hypoxia-inducible factor-signaling cascade) initiate in the visceral adipose tissue as dysfunctional responses of the adipocytes [5,6,7,8,9]. Following these processes, the adipose tissue in obese patients expresses elevated amounts of proinflammatory cytokines such as C-reactive protein, tumor necrosis factor-α, interleukin (IL)-6, and transforming growth factor (TGF)-β1. Monocyte chemotactic protein (MCP)-1 is a well-known chemokine which contributes to macrophage recruitment and activation into the adipose tissue [10,11].

Human chitotriosidase (CHIT1) is an enzyme participating in innate [12] and acquired immunity [13]. Human chitotriosidase is synthesized by the activated macrophages [14] and its expression is associated with macrophage polarization to both M1 proinflammatory and M2 anti-inflammatory phenotypes [15]. A common allelic variant of the CHIT1 gene, a 24 base-pair duplication in exon 10, has a prevalence of 6 to 10% in the European population [16,17]. In patients homozygous for this mutant allele, it activates a cryptic splice site in exon 10, leading to alternate splicing and in-frame deletion of 87 nucleotides and eventually to the absence of the enzymatic activity when 4-methylumbelliferyl-chitotrioside (4-MU-3C) is used as substrate [16].

Both adipocytes and M1 proinflammatory macrophages in the adipose tissue of obese adult patients express high levels of MCP-1 [5,18], which influences the recruitment of macrophages [10]. Based on previous data, human chitotriosidase enhances the secretion of the MCP-1 in macrophages [19]. Moreover, MCP-1 expression in the visceral adipose tissue decreases the insulin-stimulated glucose uptake, thus contributing to pathological conditions associated with obesity, such as hyperinsulinemia and type 2 diabetes mellitus [20]. The aforementioned evidence highlights the importance of the visceral adipose inflammation, as expressed by the MCP-1-mediated role of chitotriosidase, in the onset of insulin resistance.

In obese children, waist circumference (WC), waist-to–hip ratio (WHR), and waist-to-height ratio (WtHR) are indicators of visceral adiposity level and are strongly associated with obesity-associated conditions, such as non-alcoholic fatty liver disease [21], arterial hypertension [22], and metabolic syndrome [23]. Moreover, they represent independent predictors of insulin resistance (IR) in this population [24].

Insulin resistance (IR), the insensitivity of insulin-dependent tissues to the hormone action [25], is a key process linking childhood obesity to cardiovascular risk factors by increasing the risk of coronary heart disease [26,27,28,29]. Insulin resistance is directly influenced by the adipokines released in adipose tissue [20,30,31,32,33]. In obese children, IR is associated with ectopic fat deposition at the hepatic, muscular, and abdominal level [34,35,36,37,38]. More specifically, the accumulation of lipids in subcutaneous fat tissue causes a transfer of free fatty acids to the visceral adipose tissue and other insulin-responsive organs, such as the skeletal muscle and liver, leading to lipotoxicity and selective IR [39,40]. Intracellular fatty acid derivatives contribute to IR by hindering the function of the glucose transporter type 4 in the cell membrane and causing reduced glucose uptake in the skeletal muscle, while enhancing glucose production via gluconeogenesis in the liver [41].

Two surrogate indexes of IR, namely the homeostatic model assessment for insulin resistance (HOMA-IR) [42,43] and triglyceride-to-high density lipoprotein cholesterol ratio (TG/HDLc)—a non-insulin- and non-glucose-derived measure of β-cell function—have been correlated to the IR level as measured by the hyperinsulinemic-euglycemic clamp—considered the gold standard method—in obese children and adolescents [44,45].

We hypothesized that CHIT1 circulating activity is a mediator for the relationship between visceral adipose accumulation and insulin resistance in children with obesity. Thus, our study aimed to evaluate whether human circulating chitotriosidase activity, as an indicator of macrophage activation, is associated with changes in abdominal fat deposition (waist circumference, waist-to-hip ratio, and waist-to-height ratio) and IR (HOMA-IR and TG/HDLc) in children with obesity.

## 2. Materials and Methods

The study had the approval of the Iuliu Hațieganu University of Medicine and Pharmacy Ethics Committee (approval no. 179/30.05.2019). At the enrollment, children assented to participation after being provided with the information adapted to their age category (5 to 11 and 12 to 18 years old). Furthermore, their legal representatives signed a written informed consent form.

### 2.1. Patients and Clinical Variables

We conducted a prospective longitudinal exploratory study with consecutive data collection from October 2019 to July 2020 in the Endocrinology Department of the Infectious Disease Hospital in Cluj-Napoca, Romania. The eligible patients were obese children aged from 5 to 17 years who were receiving outpatient care. We included all patients previously enrolled in our cross-sectional study [46], who came for a follow-up medical evaluation in our clinic until July 2020. Obesity was defined according to the 2007 WHO recommendations as a BMI-for-age and sex corresponding to a percentile above 95 [47]. We excluded children presenting with monogenic obesity or clinical features of syndromic obesity, such as dysmorphic features, cognitive delay, or visceral malformations, from the analysis. Patients with acute infections or chronic inflammatory conditions, with metabolic syndrome at the baseline evaluation or under treatment affecting weight at baseline or follow-up, as well as those homozygous for dup24 with no circulating CHIT1 activity, were also excluded. All children received recommendations according to the medical protocol regarding basic lifestyle, eating, and physical activity (including hypocaloric diet, duration of daily physical exercises, sleep, and screen time duration adapted for the patient age) from the same trained endocrinologist during a 20-min medical consultation. Given the exploratory design of our analysis, the characteristics of the lifestyle, eating, and physical activity recommendations were beyond the study’s scope.

The anthropometric assessment at the baseline and follow-up evaluation consisted of weight, height, abdominal circumference, and hip circumference measurement after a minimum 8-hour fasting period in light clothes and barefoot (with socks). The body measurements were taken at the baseline evaluation and during the follow-up visit by the same trained endocrinologist. We used a stadiometer and a digital weight scale (Beurer, Germany) with an accuracy of ±0.1 cm for height and ±0.1 kg for weight measurements. Hip circumference (cm) was measured at the level of the widest point around the greater trochanter without trousers, and the abdominal circumference was measured at the level of the umbilicus (body tape measure, GIMA, Gessate, MI, Italy). We included in the analysis two indicators of abdominal adiposity: waist-to-hip ratio (defined as waist circumference (cm) divided by hip circumference (cm)) and waist-to-height ratio (defined as waist circumference (cm) divided by height (m)). The BMI-for-age and -sex z score was calculated using the Anthroplus application v1.0.4 provided by the World Health Organization (WHO) [47].

A trained endocrinologist classified children as prepubertal (Tanner stage 1), pubertal (Tanner stages 2–4), and postpubertal (Tanner stage 5), according to the Marshall and Tanner staging [48,49].

Two surrogate indicators for insulin resistance were included in our analysis, triglyceride-to-HDLc ratio with fasting triglycerides and high-density lipoprotein-cholesterol levels expressed in milligrams per decaliter and the Homeostatic Model Assessment for Insulin Resistance (HOMA-IR) index, which we calculated according to the formula: HOMA-IR = (fasting insulin concentration(µU/mL) × fasting glycaemia (mg/dL))/405 [50]. The triglycerides, high-density lipoprotein-cholesterol, and glycaemia levels were measured via spectrophotometry on a KONELAB 60i P2 (Thermo, Vantaa, Finland), and insulin levels were evaluated using chemiluminescence on a Mindray CL-1200i analyzer (Mindray, Shenzhen, China).

### 2.2. Chitotriosidase Circulating Activity

Chitotriosidase plasma activity was evaluated by a fluorometric method, according to Hollak et al. [51], by using 4-methylumbelliferyl-chitotrioside as substrate and was expressed as nanomoles of hydrolyzed substrate per milliliter per hour (nmol/mL/h). Our laboratory had a reference range of values between 3 and 100 nmol/mL/h.

### 2.3. Genetic Analysis of CHIT1 Gene

Specific primers (forward primer 5′-GAAGAGGTAGCCAGGCTCTGG-3′ and reverse primer 5′-CTGCCGTAGCGTCTGGATGAG-3′) were used to amplify the fragments of 195 and 219 base pairs in exon 10 of the CHIT1 gene. Our protocol for dup24 identification included polymerase chain reaction (PCR) followed by electrophoretic separation. The genotypes were confirmed by the Sanger sequencing method by using the same set of primers. The detailed protocol of the aforementioned methods was described in our previously reported article [46].

### 2.4. Statistical Analysis

Statistical analysis was performed in the STATISTICA program (Version 13.5, StatSoft, Tulsa, OK, USA). Graphical representations were obtained using the Seaborn program (Version 0.9.0, Python library, M. Waskom). Categorical variables are expressed as absolute frequency and percentage (no., %), whereas quantitative variables are summarized as median, interquartile range (IQR, [percentile 25 to percentile 75] and {minimum to maximum values} and mean and standard deviation. Spearman’s rank correlation coefficient (ρ) was used to evaluate the monotonic correlation between quantitative variables in cross-sectional analyses at the baseline and follow-up examinations. The McNemar test with continuity correction was used for longitudinal analyses to test the bivariate associations between qualitative variables. The Wilcoxon signed-rank test was performed to evaluate differences in quantitative variables and the Mann–Whitney test was used to evaluate the differences between independent groups.

All statistical tests used in data analysis were two-sided with a significant result achieved whenever *p*-value < 0.05.

## 3. Results

### 3.1. Description of the Study Sample and Longitudinal Analysis

We included 29 patients (16 girls and 13 boys) in the study, with a mean age of 11.36 ± 3.61 years at the initial evaluation. The median follow-up period was 7 months (IQR, [5 to 8.5]) and varied from 2 to 13 months.

**Figure 1:**
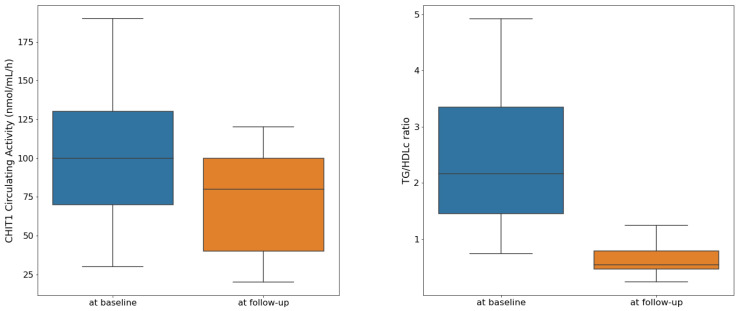
Decrease in CHIT1 circulating activity and TG/HDLc at follow-up compared with baseline.

The CHIT1 circulating activity significantly decreased at the follow-up visit, with a baseline median value of 100 nmol/mL/h, (IQR [70 to 130] and {30 to 320} nmol/mL/h) versus a follow-up median value of 80 nmol/mL/h, (IQR [40 to 105] and {20 to 300} nmol/mL/h), (Wilcoxon test, *p* = 0.015, see Figure 1). The TG/HDLc also significantly decreased from a median value of 2.16, (IQR [1.44 to 3.62] and {0.74 to 6.49}) to a median value of 0.54, (IQR [0.45 to 0.81] and {0.45 to 0.81}), (Wilcoxon test, *p* < 0.001, see Figure 1).

The HOMA-IR did not significantly change in our longitudinal analysis (Wilcoxon test, *p* = 0.349). The decrease in indicators of abdominal adiposity from baseline through follow-up did not reach statistical significance (Table 1).

### 3.2. Cross-Sectional Analysis at the Baseline

**Figure 2:**
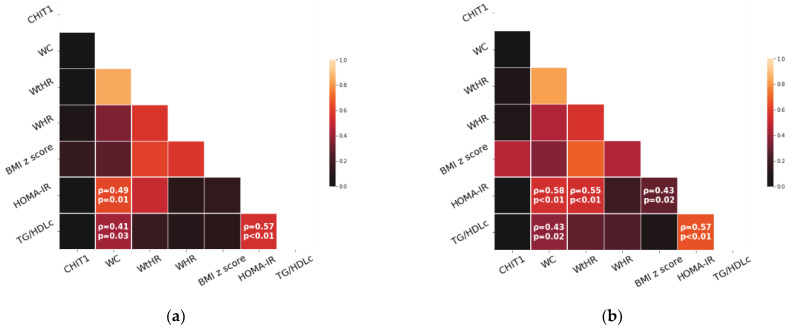
Cross-sectional associations between surrogate indexes of IR and indicators of adiposity: (**a**) at baseline and (**b**) at follow-up. The Spearman’s rank correlation coefficients (ρ) are displayed only in case of significant correlations (*p* < 0.05).

Age was positively correlated with HOMA-IR (Spearman’s rank correlation coefficient, ρ = 0.49, *p* = 0.01) and with TG/HDLc (Spearman’s rank correlation coefficient, ρ = 0.41, *p* = 0.03). Considering the relationship between the indicators of abdominal adiposity and the IR, WC was the sole indicator positively correlated to both surrogate indexes of IR, namely to HOMA-IR (Spearman’s rank correlation coefficient, see Figure 2) and to TG/HDLc (Spearman’s rank correlation coefficient, see Figure 2). Cross-sectional analysis also revealed a positive correlation between HOMA-IR and TG/HDLc (Spearman’s rank correlation coefficient, see Figure 2). Partial correlation showed a positive correlation between HOMA-IR and TG/HDLc (partial correlation coefficient = 0.44, *p* = 0.02) and between WC and HOMA-IR (partial correlation coefficient = 0.47, *p* = 0.02), while controlling for the effect of age.

At baseline, chitotriosidase circulating activity was not associated with the indicators of adiposity (WC, WHR, WtHR, or BMI-for-age z score) or the surrogate indexes of IR (*p*-values > 0.05).

### 3.3. Cross-Sectional Analysis at the Follow-Up

Considering the relationship between the indicators of adiposity and the IR, WC, as well as the waist-to-height ratio and BMI-for-age z score, were positively correlated with HOMA-IR (Spearman’s rank correlation coefficients, see Figure 2). WC was also positively associated with TG/HDLc (Spearman’s rank correlation coefficient, see Figure 2).

Regarding the surrogate indexes of IR, HOMA-IR was correlated with TG/HDLc (Spearman’s rank correlation coefficients, Figure 2) at follow-up. We found no correlation between age and the surrogate markers of IR at the follow-up.

### 3.4. Influence of dup24 in the CHIT1 Gene upon the Change of CHIT Circulating Activity

In our sample, five patients (17.24%) were heterozygous for dup24. When stratifying the patients according to the presence of the dup24 allele, we found no significant difference in the CHIT1 circulating activity in heterozygous patients and the patients without the allele at baseline (Mann–Whitney test, *p* > 0.05).

## 4. Discussions

### 4.1. CHIT1 Circulating Activity and the Insulin Resistance Expressed by TG/HDLc

Our longitudinal analysis found that a significant reduction in the CHIT1 circulating activity co-occurs with a decrease in the insulin resistance, as expressed by the TG/HDLc, in children with obesity (Figure 1). Our finding aligns with previous data indicating an inter-relation between chronic inflammation and insulin resistance in children with obesity [52]. Singer and Lumeng demonstrated that insulin resistance emerges from obesity-driven inflammation as early as childhood [53]. In pediatric patients with obesity, a decreased insulin sensitivity was associated with the activation of the inflammasomes–cytosolic protein complexes involved in innate immune system activation [54]. Inflammation within the visceral adipose tissue negatively affects adipocyte insulin sensitivity and may further trigger systemic insulin resistance via macrophage-mediated actions rather than via lipid overload [55]. In reverse, insulin resistance enhances macrophage accumulation and cytokine production [56].

Macrophages constitute the predominant cell subtype in the adipose tissue of obese children and adults [57,58], which contribute to the crown-like structures around apoptotic adipocytes [59]. Following proliferation, adipose tissue macrophages can change their anti-inflammatory M2 phenotype from normal conditions (i.e., adults without obesity) to the M1 proinflammatory status (i.e., obese adults). M1 macrophages release type 1 cytokines, such as TNF-α, IL-1β, IL-6, and MCP-1 [60,61,62,63]. The above cytokines induce insulin resistance via activation of the intracellular signaling pathways [64].

Human chitotriosidase is released from the activated macrophages and its local overexpression further promotes macrophage recruitment and the synthesis of MCP-1 [65]. The mediating role of MCP-1 between the accumulation of macrophages in the adipose tissue and insulin resistance might require an increase in CHIT1 synthesis and its circulating activity [56]. Further research should investigate the molecular mechanisms of insulin resistance concerning the role of CHIT1 and MCP-1 in obese children. Based on clinical research suggesting that CHIT1 indicates the subclinical inflammation in atherosclerosis, non-alcoholic fatty liver disease, and impaired glucose tolerance in animal models and humans (i.e., children and adults) [66,67,68], our findings open a new avenue of biomarkers research in metabolic complications of childhood obesity.

Concerning the surrogate index of insulin resistance, previous studies showed that TG/HDLc is associated with the hyperinsulinemic-euglycemic clamp—the gold standard measure of insulin sensitivity—and with a highly specific oral glucose tolerance test (i.e., whole-body insulin sensitivity index) in obese adolescents [45]. Similarly, our cross-sectional evaluations at baseline and follow-up found a positive association between TG/HDLc and HOMA-IR, irrespective of the influence of age (Figure 2). The role of the TG/HDLc in assessing insulin resistance has been explained by the increased delivery of free fatty acids to non-adipose tissues, mainly the liver and muscle [69,70]. The resistance to the antilipolytic effect of insulin over the number of triglycerides in excess, together with the de novo synthesis of free fatty acids in the liver, results in a progressive increase in free fatty acids and subsequent dyslipidemia [71]. An increase in the TG/HDLc predicts a rise in the cardiometabolic risk in children [72,73,74,75], with a value of ≥2.2 being proposed as an accurate screening parameter for insulin resistance, arterial stiffness, and metabolic syndrome in obese children [76].

In addition to being an indicator of insulin resistance, TG/HDLc also has the capacity to reflect the extent of dyslipidemia in children with obesity [45]. The Third National Health and Nutrition Examination Survey revealed that dyslipidemia, consisting mainly of low HDL cholesterol levels (40–50% of adolescents) and high TG (25–30% of adolescents), was the most common feature of the metabolic syndrome in obese adolescents [77]. Moreover, dyslipidemia is part of the cluster of cardiovascular risk predictors, also including central obesity, hyperglycemia, and elevated blood pressure, according to the majority of definitions of the pediatric metabolic syndrome [78,79,80]. The IDEFICS (Identification and prevention of Dietary- and Lifestyle-induced health Effects In Children and Infants) study proposed a consensual definition including insulin resistance as expressed by HOMA-IR value together with dyslipidemia as reflected by the triglycerides and the high-density lipoprotein cholesterol level, waist circumference, and systolic and diastolic blood pressures [81].

Thus, the dual role of TG/HDLc lies in its capacity to reflect both insulin resistance and anomalies of lipid metabolism [45]. The significant association of TG/HDLc with CHIT1 activity might indicate the capacity of CHIT1 circulating activity in reflecting both dyslipidemia and insulin resistance in the context of visceral tissue inflammation. This role might have important implications in defining more accurately the risk of metabolic syndrome in children.

### 4.2. Relationship between the Clinical Indicators of Adiposity and Insulin Resistance

Our cross-sectional analyses found a significant positive association between the extent of adiposity, particularly abdominal adiposity as expressed by the WC and WtHR, and the surrogate indexes of insulin resistance (Figure 2). The decrease in CHIT1 activity at follow-up co-occurred with the reduction of all indicators of abdominal adiposity, namely WC, WHR, and WtHR, without reaching statistical significance (Table 1). This finding highlights that the abdominal adiposity has a greater impact on insulin resistance than the subcutaneous adipose tissue (BMI-for-age z score) and this influence is mediated by subclinical inflammation (as reflected in CHIT1 activity).

However, abdominal obesity is determined by the accumulation of both intra-abdominal fat depots or visceral fat and subcutaneous abdominal tissue [82]. Still, our findings are aligned with those of previous research that showed that the visceral adiposity confers a higher cardiometabolic risk in adults and children [83,84] than the subcutaneous adipose tissue mass [85,86], and this relationship is mediated by macrophage-mediated inflammation [5,87].

In contrast with WC, WHR, and WtHR, which indicate the extent of abdominal obesity, the BMI-for-age z score reflects in particular the subcutaneous fat distribution [88]. In our longitudinally assessed patients, no significant change in BMI-for-age z score was found at the follow-up evaluation. Similarly, Hardy et al. showed that BMI-for-age z score was not associated with inflammation progression [89], while Sinaiko et al. found that insulin resistance and BMI-for-age z score were independent predictors of cardiovascular risk [90].

Given the established role of visceral adipose tissue as the starting point of inflammation in contrast with subcutaneous fat, our findings might indicate that CHIT1 is a sensitive marker of the variation in subclinical inflammation grade even before indicators of the abdominal adiposity change. The initial state of overnutrition at the onset of obesity precedes subclinical inflammation [54], but the temporal relationship between inflammation and adiposity is less evident in more advanced phases of obesity [91].

### 4.3. The Influence of Puberty on Insulin Resistance

Our study longitudinally assessed obese children from prepuberty through late puberty. The puberty staging at the baseline did not significantly progress through follow-up (Table 1). Consistent data showed that puberty leads to a rise in the insulin concentration that compensates for a decrease in the insulin sensitivity of approximately 30–50%, irrespective of the changes in body fat percentage [92,93]. Marwitz et al. [94] showed that insulin sensitivity decreases particularly from prepuberty (Tanner stage I) through late puberty (Tanner stage IV).

### 4.4. Limitations and Further Research

The exploratory design, small sample size, and the lack of a control group affect the generalizability of our results. The relatively short follow-up period limited the likelihood of observing significant changes in BMI-for-age z score and the indicators of abdominal obesity. In the same manner, a more extended period might better define the role of the heterozygosity for dup24 in the CHIT1 gene for the variability in the enzyme circulating activity. A standardized intervention for weight loss might also be useful in reaching significant clinical changes of the indicators of adiposity.

The inclusion in our analysis of patients formerly evaluated in a cross-sectional study [46] might have negatively influenced the prospective hypotheses of our study.

In our study, we used only clinical indicators of abdominal obesity, which may not reflect accurately the extent of visceral abdominal adiposity in children. More precise measures for fat distribution and expansion, such as magnetic resonance imaging and computer tomography of the abdominal mass [95], together with dual X-ray absorptiometry [96], might provide a more precise estimation of the relationship between inflammation and visceral adiposity. Since 2021, attempts have been made to develop novel indicators that combine clinical and biological features to better evaluate the cardiovascular risk in children with obesity [97].

Concerning the assessment of insulin sensitivity, gold standard techniques, such as a hyperinsulinemic-euglycemic clamp, might capture more accurately the relationship between CHIT1 circulating activity and insulin resistance in children with obesity.

## 5. Conclusions

Human chitotriosidase might be a sensitive indicator of low-grade inflammation in the visceral adipose tissue in children with obesity. Its variation might precede the progression of abdominal adiposity and might reflect the inflammation-mediated insulin resistance in the pediatric population. In the clinical setting, chitotriosidase might help in detecting obese children with a high risk of metabolic syndrome in need of a closer follow-up intervention. Thus, our preliminary results are worth being investigated in larger cohorts.

## Figures and Tables

**Table 1:** Demographic and clinical characteristics of the evaluated children.

Characteristic	At Baseline	At Follow-Up	*p*-Value
Age, years	11.36 ± 3.61	11.95 ± 3.6	<0.001
	11.42 [8.83 to 13.33]	12.08 [9.67 to 14.17]	
	{5.08 to 18.42}	{5.5 to 18.92}	
Tanner staging			0.264 *
prepubertal	14 (48.28)	8 (30.77)	
pubertal	15 (51.72)	18 (69.23)	
BMI-for-age z score	3.01 ± 1.09	2.9 ± 0.94	0.127
	2.57 [2.25 to 3.78]	2.67 [2.34 to 3.32]	
	{1.82 to 6.32}	{1.86 to 5.86}	
Waist circumference, cm	91.79 ± 16.78	91.11 ± 15.16	0.331
	91 [80 to 98]	90 [80 to 97.5]	
	{64 to 136}	{63 to 130}	
Waist-to-hip ratio	0.96 ± 0.06	0.95 ± 0.05	0.327
	0.95 [0.91 to 1.01]	0.95 [0.90 to 0.98]	
	{0.83 to 1.05}	{0.87 to 1.06}	
Waist-to-height ratio	0.61 ± 0.06	0.6 ± 0.06	0.145
	0.61 [0.57 to 0.65]	0.59 [0.55 to 0.64]	
	{0.48 to 0.8}	{0.49 to 0.72}	

Data are presented as absolute (relative) frequencies or arithmetic mean ± standard deviation and median [Q1 to Q3] and {min to max}; *p*-values obtained from McNemar test (qualitative data *) or Wilcoxon test (quantitative data); significant *p*-value.

## Data Availability

The raw data analyzed in this study are part of a Ph.D. study and can be obtained upon reasonable request addressed to Ioana Țaranu (taranu.ioana@umfcluj.ro).
